# The plant-based immunomodulator curcumin as a potential candidate for the development of an adjunctive therapy for cerebral malaria

**DOI:** 10.1186/1475-2875-10-S1-S10

**Published:** 2011-03-15

**Authors:** Patrice N Mimche, Donatella Taramelli, Livia Vivas

**Affiliations:** 1 Faculty of Infectious and Tropical Diseases, Department of Immunology and Infection, London School of Hygiene and Tropical Medicine, Keppel St, London WC1E 7HT, UK; 2 Dipartimento di Sanità Pubblica -Microbiologia-Virologia, Università di Milano, Via Carlo Pascal 36, 20133 Milano, Italy

## Abstract

The clinical manifestations of cerebral malaria (CM) are well correlated with underlying major pathophysiological events occurring during an acute malaria infection, the most important of which, is the adherence of parasitized erythrocytes to endothelial cells ultimately leading to sequestration and obstruction of brain capillaries. The consequent reduction in blood flow, leads to cerebral hypoxia, localized inflammation and release of neurotoxic molecules and inflammatory cytokines by the endothelium. The pharmacological regulation of these immunopathological processes by immunomodulatory molecules may potentially benefit the management of this severe complication. Adjunctive therapy of CM patients with an appropriate immunomodulatory compound possessing even moderate anti-malarial activity with the capacity to down regulate excess production of proinflammatory cytokines and expression of adhesion molecules, could potentially reverse cytoadherence, improve survival and prevent neurological sequelae. Current major drug discovery programmes are mainly focused on novel parasite targets and mechanisms of action. However, the discovery of compounds targeting the host remains a largely unexplored but attractive area of drug discovery research for the treatment of CM. This review discusses the properties of the plant immune-modifier curcumin and its potential as an adjunctive therapy for the management of this complication.

## Background

Malaria, a disease caused by the Apicomplexan parasite of the genus *Plasmodium*, remains a serious public health threat in tropical and sub-tropical countries. It is the most important parasitic disease in humans with 300 to 500 million infected individuals every year. A proportion of these patients, mostly children under five, is at risk of developing severe anaemia or cerebral malaria (CM), which claims at least 1 million lives annually [1]. CM is a life-threatening complication of this infection which often occurs in non-immune individuals and those in which standard anti-malarial treatment has failed [2]. The effectiveness of anti-malarial treatment has been hampered by the emergence over the years of parasites resistant to almost all available anti-malarial drugs, including more lately the artemisinin class of drugs [3]. The reduced susceptibility to artemisinin derivatives recently identified in parasite isolates from SE Asia may compromise their efficacy for the treatment of CM, which currently relies on artesunate or quinine [4], and is a worrying sign, as resistance may spread quickly to other parts of the world. Treatment failure of uncomplicated cases will inevitably increase the number of patients susceptible to develop severe complications. Therefore, there is an urgent need to develop new anti-malarial drugs and drug combinations or alternative strategies that circumvent the development of parasite drug resistance with the final aim of reducing the burden of this disease. These approaches may involve the identification of immune-modifying compounds with the capacity of enhancing macrophage’s phagocytic activity, resulting in a reduction of the parasite biomass [5], down-regulating excessive pro-inflammatory type 1 response, and reducing the expression of adhesion molecules and subsequent sequestration of parasitized erythrocytes (PE) (a hallmark of human CM) in the cerebral microvasculature, thus improving survival of CM patients. Targeting the host, using immunomodulatory compounds, might be a useful strategy to complement the direct anti-parasitic activity of standard anti-malarial drugs and, as such, present a valuable tool in the management of CM and limit the emergence of resistant parasites. This review highlights the potential benefits that the plant-based natural product curcumin may have as an immunomodulator and as an adjunctive therapy for CM.

### Rationale for the use of immunomodulators for the treatment of cerebral malaria

Cerebral malaria, one of the most severe complications of *Plasmodium falciparum* infections, is associated with various pathophysiological processes [6]. CM is mostly characterized by hyper-parasitaemia and by an excessive production of type 1 pro-inflammatory cytokines followed by up-regulation of endothelial cell adhesion molecule expression which contributes to the sequestration of PE in the brain microvasculature [7]. Understanding the molecular events implicated in the onset of CM would pave the way for the development of adjunctive therapies that may reduce cerebral damage by modulation of the pathological processes involved in its development, and thus prevent subsequent mortality and neurological sequalae. The association of immunomodulators with anti-malarial drugs could prove to be beneficial for the management of this condition [8]. This approach has already been tested using a variety of compounds in animal models of experimental cerebral malaria (ECM) [9-13] as well as in humans [14,15]. One of the strategies currently being investigated is to target the peroxisome proliferator activated receptor gamma (PPARγ), a nuclear receptor involved in the regulation of the scavenger receptor CD36, which mediates non-opsonic phagocytosis of PE [16]. Pharmacological upregulation of CD36 in monocytes/macrophages by PPARγ agonists increases CD36 dependant phagocytosis of PE *in vitro*[17]. In a murine model of malaria, administration of the PPARγ agonist rosiglitazone, to *Plasmodium chabaudi*-infected mice, significantly decreased parasitaemia levels in wild type compared to CD36 knock-out mice and improved survival in *Plasmodium berghei*-ANKA infected groups [12]. These findings warranted a randomized, double-blind, placebo-controlled trial to assess the efficacy of rosiglitazone, as an adjunctive therapy for the treatment of *P. falciparum* malaria. That trial showed a reduction in parasite clearance time and inflammatory markers in patients with uncomplicated malaria under a treatment regimen consisting of atovaquone + rosiglitazone compared to patients treated with atovaquone + placebo [18]. This evidence, together with a recent genome-wide association study linking a locus containing *PPARγ* with improved survival in a rodent malaria model [19], led us to speculate that pharmacologically targeting the signalling pathways involved in PPARγ/CD36 expression during a malarial infection might improve CM treatment outcome.

Erythropoietin (Epo), a hormone produced by the kidneys which modulates the survival of developing erythroid precursors and production of new erythrocytes in the bone marrow, has been explored for the management of CM in animals and humans [9]. In the *P. berghei*-ANKA murine model of ECM, injection of high doses of Epo at the beginning of symptoms, significantly reduced the expression of pro-inflammatory cytokines (TNF and Interferon-γ) and improved the survival of mice with ECM compared to untreated mice [20]. Furthermore, in the same murine model of ECM, the doses of Epo were decreased six fold and its administration combined with artesunate was delayed to the sixth day post-infection. The results indicated that the survival rate was higher in mice receiving the combination Epo-artesunate than in mice treated with artesunate alone [21]. Clinical evidence for a neuroprotective role of Epo in humans emerged from a study of African children with CM in which high plasma levels of Epo were associated with a 70% reduction of the risk of being discharged with neurological sequelae [22]. These findings provided preliminary evidence for a clinical trial assessing the safety of Epo as an adjunctive therapy for children with CM in Mali. In this trial, the administration of high doses of Epo in CM children did not result in any side effects when evaluated on a short-term basis, and no significant increase in the case fatality rate of the combined Epo-quinine administration was observed [15].

Activated charcoal is also being explored as a potential adjunctive therapy for the treatment of CM. Oral activated charcoal (oAC) is highly effective at adsorbing a range of endotoxin-induced cytokines from the bloodstream including TNF, IL-1 and IL6 [23,24]. It was recently found that oAC protected mice against ECM and more importantly did not interfere with the pharmacokinetics of parenteral artesunate in humans [10]. The fact that oAC is a safe and well-tolerated compound already used in the clinic may accelerate its development as an adjunctive therapy for CM. Alternatively, the Rho-Kinase inhibitor, Fasudil, a drug approved for human use for cardio- and neuro-vascular diseases, is being proposed as an adjunctive therapy for severe malaria. Fasudil, which has anti-apoptogenic properties, protects and restores *in vitro*, the damage to the endothelial barrier resulting from PE adhesion [25,26]. It is worth mentioning that the adhesion of PE to human endothelial cells activates Rho Kinases and caspases leading to apoptosis [27]. Furthermore, Fasudil also modulates CM in *P. berghei-*ANKA infected mice [13]. PE-induced oxidative stress on endothelial cells is also involved in the pathology of CM and supplementation with anti-oxidant compounds protected endothelial cells *in vitro*[*28*]. In addition, treatment of *P. berghei*-infected mice with the antioxidant N-acetylcysteine (NAC) or the iron chelator, deferoxamine, in combination with chloroquine at the first signs of CM, prevented the development of persistent cognitive dysfunction in infected mice [11]. However, a clinical trial of NAC in combination with artesunate as adjunct therapy failed to detect any benefit on outcome in patients with severe *P. falciparum* malaria [14]. Although all these treatment options for adjunctive therapies in CM seem promising (for a detailed review of adjunctive therapies for malaria see [8, 29-30]), none of these compounds possess specific anti-malarial activity on their own. Therefore, plant-based immunomodulators displaying dual anti-malarial and immunodulatory mechanisms of action could become ideal candidates for anti-malarial drug development. This strategy has been explored with the natural product curcumin which shows immunomodulatory properties and has been found to prevent death from CM in *P. berghei* infected mice [13].

### Curcumin, a plant-based immunomodulator

For thousands of years, some of the most effective anti-malarial drugs have been derived from plants. Quinine and artemisinin, the only two molecules of choice for the treatment of severe malaria were isolated from the bark of the cinchona tree and the Chinese plant *Artemisia annua*, respectively. Although artemisinin and its derivatives have had a major impact on treatment when given in combination with other anti-malarials, they are limited by their short half-life, cost and safety concerns in pregnant women [31]. Poor distribution and access to these drugs among the population living in malaria endemic areas, means that many still rely on numerous herbal preparations or diets from traditional healers for the management of fever and malaria [32].

**Figure 1:**
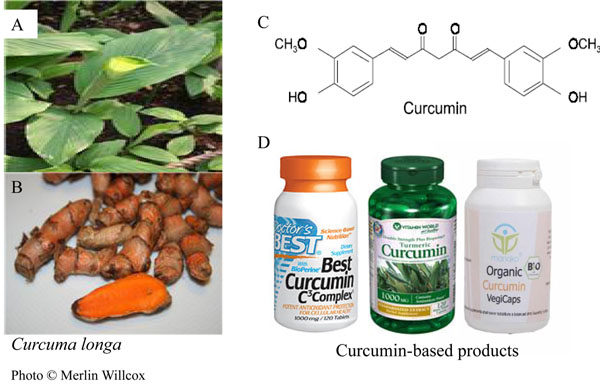
**Chemical structure of curcumin.** Curcumin (diferuloylmethane) is the active principle of the perennial herb *Curcuma longa* also known as turmeric (A) and is extracted from its roots (B). Molecular structure of curcumin (C). Curcumin-based products are available as dietary supplements (D). *Curcuma longa* photos © Merlin Willcox.

The natural product curcumin (1,7-bis(4-hydroxy 3-methoxy phenyl)-1,6-heptadiene-3,5-dione) is a polyphenolic compound extracted from the rhizome of *Curcuma longa L.* (family Zingiberaceae) (Figure 1) commonly used in the Asian sub-continent, especially in India, as a dietary spice to provide colour and flavour [33]. In traditional Indian medicine (*Ayurveda*), curcumin has been considered an effective drug for the treatment of various disorders, and recent studies have substantiated and provided scientific evidence regarding its prophylactic and therapeutic potential, unravelling the anti-inflammatory, anti-carcinogenic, and anti-infectious activities of this natural product (Reviewed in [33-35]).

### Curcumin for malaria therapy?

Studies carried out both *in vitro* and *in vivo* indicated that curcumin possesses a moderate anti-malarial activity with an IC_50_ ranging from 5-10µM *in vitro*[*36*,*37*], [38]. In order to expand its potential as anti-malarial, curcumin derivatives have been synthesized and some of them demonstrated an increased anti-malarial activity *in vitro* with an IC_50_ of ~400nM [39]. It was then demonstrated that this compound inhibits histone acetyltransferase (HAT) and increases the production of reactive oxygen species (ROS) in the malaria parasite [36]. Despite its moderate anti-malarial activity, other investigators suggested that the mechanism of action of curcumin might mirror that of artemisinin. In fact, artemisinin derivatives seem to target the parasite mitochondria and the flavin co-factors thus increasing the production of ROS. This reactivity is dependent on the endoperoxide bridge, which is essential for the anti-malarial activity of artemisinins [40,41]. Curcumin seems to be more effective in killing the malaria parasite at the trophozoite stage while artemisinins display broad activity against all stages of the intraerythrocytic development of the malaria parasite [38]. Molecular docking experiments showed that curcumin can efficiently bind the malaria sarco-endoplasmic reticulum calcium ATPase (SERCA-PfATPase6) [42,43] an ATP coupled Ca^2+^ ion pump involved in metabolic arrest which was first thought to be the biological target of artemisinin [44-46]. Interestingly, oral administration of curcumin followed by a single injection of the artemisinin derivative α-β-arteether to *P. berghei*-infected mice, prevented recrudescence usually associated with α-β-arteether monotherapy and ensured almost 100% survival of animals [47]. By contrast, a subsequent study showed that although curcumin had modest anti-malarial efficacy (delay in the peak parasitaemia) in mice, it was not able to modify the course of infection, when administered with artemisinin nor to reverse a *P. chabaudi* artemisinin-resistant phenotype [48]. The modest anti-malarial activity of curcumin in this model system was attributed to its immunomodulatory activities, which could be beneficial in delaying parasite growth. However, inherent differences between the two experimental murine models may help explain these contradicting results, since in contrast to *P berghei* -ANKA, *P chabaudi* causes a non lethal infection and does not lead to CM in CD1 mice [48].

### Curcumin as an adjunctive therapy for severe/cerebral malaria infection

#### **Curcumin as a modulator of the innate immune response to malaria infection**

The severity of malaria infections is often associated with a large parasite biomass which triggers defence mechanisms that hinder parasite multiplication and are, therefore, important contributing factors in host survival [49]. A key feature of curcumin is that it possesses both anti-oxidant and pro-oxidant activities [50,51]. Available evidence supporting a protective role of ROS in malaria has emerged from animal and human studies. In humans, the elevated production of oxygen radicals was associated with faster parasite clearance in children with uncomplicated *P. falciparum* malaria [52]. Furthermore, in the athymic or *scid/bg* mouse model of malaria, a pro-oxidant vitamin E-deficient diet enriched with fish oil suppressed a lethal *P. yoelii* infection [53]. Although the actual molecular events leading to protection from malaria in mice fed this pro-oxidant diet were not elucidated, an enhanced pro-oxidant activity in these animals could have either directly killed the parasites or activated signalling pathways that could have contributed to the killing of PE via non-opsonic phagocytosis. In fact, ROS increase the expression of the scavenger receptor CD36 in monocytes/macrophages [54] and CD36 mediates non-opsonic phagocytosis of PE by macrophages [16,55].

**Figure 2:**
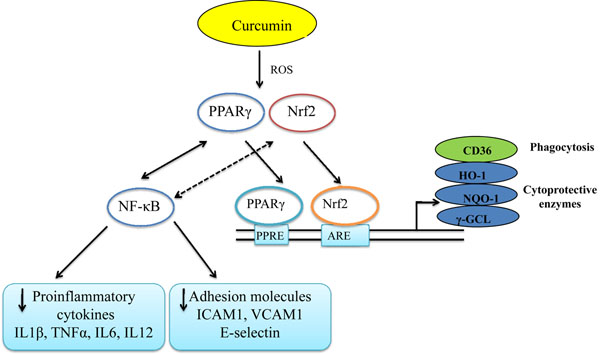
**Schematic diagram illustrating the main biological targets of curcumin that can be exploited in suppressing immunopathological events associated with cerebral malaria.** Upon exposure of cells to curcumin, a transient induction of reactive oxygen species (ROS) may occur. This potentially leads to activation of a signalling cascade involving the peroxisome proliferator activated receptor gamma (PPARγ) and induction of the redox-sensitive factor Nrf2. Activated-Nrf2 and PPARγ translocate in the nucleus, where they bind to their target genes via their respective binding sites,the anti-oxidant response element (ARE) for Nrf2 and the peroxisome proliferator response element (PPRE) for PPARγ. In monocytes/macrophages activation of Nrf2 or PPARγ will lead to upregulation of the surface expression of CD36, increasing their phagocytic activity [55]. In other cell types such as endothelial cells, activation of Nrf2 and PPARγ may lead to upregulation of cytoprotective enzymes [haem oxygenase 1 (HO-1), NADPH quinine oxidoreductase-1 (NQ-1), gamma-glutamate cysteine ligase (γ-GCL)], counteracting free radical-induced damage and exerting a neuroprotective effect [87]. On the other hand, PPARγ (solid arrows) and perhaps Nrf2 (dashed arrows) could exert their anti-inflammatory activities by inhibiting NF-κB activation, thereby downregulating proinflammatory cytokine responses and adhesion molecule expression which are all implicated in the pathology of CM.

In addition to its specific anti-malarial activity, the immunomodulatory properties of curcumin affect various cell types of the immune system [Reviewed in [56]). When administered *in vivo* to mice, a significant increase in macrophage phagocytic activity was observed [57]. Furthermore, curcumin increased non-inflammatory phagocytosis of latex beads in murine macrophages *in vitro*[58]. From the same perspective, it was demonstrated that curcumin increased the surface expression of CD36 on human monocytes/macrophages and CD36-dependent phagocytosis through a transient production of ROS which was downregulated by the anti-oxidant NAC [38]. Thus, these findings partly support the view that CD36 can be seen as a marker of the enhanced macrophage phagocytic activity observed in curcumin-treated animals [57]. CD36 expression is regulated either by activation of the nuclear receptor PPARγ or by the redox-sensitive transcription factor nuclear related {erythroid-derived 2} factor (Nrf2) [17,59]. Interestingly, these two transcription factors have been found to be activated in curcumin-treated monocytes/macrophages [60,61], though experimental evidence suggested that curcumin is not a genuine PPARγ agonist [62]. To summarize, it is likely that curcumin enhances discrete factors implicated in the innate immune response to malaria infection (e.g CD36) via a cascade of events involving transient production of ROS resulting in PPARγ/Nrf2 activation and upregulation of monocytes/macrophages CD36 surface expression and enhanced phagocytosis of PE (Figure 2).

#### Curcumin downregulates proinflammatory cytokine responses and expression of adhesion molecules on human endothelial cells in vitro

Cerebral malaria is the result of deleterious patholophysiological processes that take place in a *Plasmodium*-infected host [6]. In human malaria, a consistent histological finding in CM in both children and adults is the presence of infected and non-infected erythrocytes packed within cerebral microvessels [63]. This sequestration of PE and non-infected erythrocytes reduces the microvascular flow thereby causing disruption of blood brain barrier, cerebral oedema and tissue hypoxia [30, 63-64]. In addition, the release of neurotoxic and inflammatory mediators that may leak across the blood-brain barrier and cause more damage to the microvascular endothelium also contribute to this pathology (reviewed in [6]). The sequestration process is the result of increased expression of cytoadhesion molecules mainly ICAM1, VCAM1 and E-selectin on brain endothelial cells stimulated by the overproduction of inflammatory cytokines [65] or by the direct adhesion of *P. falciparum* to endothelial cells [66]. In fact, adhesion of *P. falciparum* to human brain endothelial cells *in vitro* creates an inflammatory environment via stimulation of NF-κB which in turn upregulates expression of ICAM1 [66,67]. Most of the adjunctive therapies for the management of CM have been tested in mice and careful precautions should be taken when extrapolating findings from animal models to human CM [68]. However, some of the key features of human CM can also be replicated in the *P. berghei* model as discussed by Riley at al [69], making it reasonably suitable for preliminary evaluation of potential CM adjunctive therapies.

Curcumin exhibits profound anti-inflammatory activities due to inhibition of NF-κB activation [70]. This can explain the rationale for the use of curcumin in Ayurveda as a treatment for chronic inflammatory diseases [71]. This immunomodulatory property of curcumin may be useful in protecting the brain endothelium from the damages caused by the sequestration of PE. Curcumin administered in conjunction with the standard treatment for CM, artesunate or quinine, might contribute to the reversal of parasite sequestration and inflamamtion, reducing the risk of neurological sequaelae. Interestingly, administration of curcumin (50mg/kg, twice a day for 6 days post infection) to C57Bl/6 mice infected with *P. berghei*-ANKA prevented CM and delayed death by 10 days [13]. In addition, it has been shown *in vitro* that curcumin reduces the production of proinflammatory cytokines TNF, IL12p40 and IL6 in PBMC primed with trophozoites/schizonts stages of *P. falciparum* and downregulated the expression of ICAM1, VCAM1 and E-selectin in TNF-activated human endothelial cells [38]. The down regulation of proinflammatory cytokines and adhesion molecules observed following PBMC exposure to curcumin *in vitro*, is perhaps the result of Nrf2 or PPARγ activation which exert anti-inflammatory actions by blocking NF-κB activation (Figure 2) [72]. In fact, in an animal model of traumatic brain injury it was observed that Nrf2 deficient mice have enhanced NF-κB activation, inflammatory cytokine production and increased ICAM-1 expression in the brain compared to their wild type counterparts [73]. Furthermore, induction of haem oxygenase 1 (HO-1) which is a downstream target of the Nrf2 activation cascade (Figure 2) suppressed the pathology of ECM in mice [74] while activating Nrf2 pathway was demonstrated to be a potential therapeutic target in brain inflammation [75]. Curcumin inhibits the adhesion of thrombin-activated platelets to brain microvascular endothelial cells *in vitro*[76] which are thought to accumulate in the brain microvasculature in murine and paediatric CM patients [77]. Recent genome wide analysis studies of inbred mouse lines confirming the important role that *PPARγ* might have in malarial survival [19], support a therapeutic approach in which the targeting of this transcription factor (using curcumin for example) could be useful in the management of CM. In addition to PPARγ, it might be worth investigating the effect of targeted deletion of Nrf2 in brain microvascular endothelial cells *in vitro* as well as *in vivo* in order to validate the Nrf2 activation pathway as an additional therapeutic target for CM.

In malaria endemic areas, both severe malaria and sepsis may often occur together [78]. In an animal model of sepsis, disruption of Nrf2 was associated with a significant increase in proinflammatory cytokines and mortality in response to endotoxin-induced septic shock [79]. Management of severe malaria and sepsis could be supported with anti-inflammatory agents that may target Nrf2, such as curcumin, in combination with potent artemisinin derivatives. In fact, artemisinins also possess significant anti-inflammatory activity suppressing TNFα and IL-6 in a murine model of sepsis [80]. Moreover, the artemether-lumefantrine combination has been found to decrease mortality from sepsis in Ugandan children without malaria [81]. In a recent multicentre, open label, randomised trial comparing artesunate versus quinine (Aquamat Study) for the treatment of severe *P. falciparum* malaria in African children, intravenous artesunate has now been declared to be the most effective treatment for severe malaria [4]. This gives support to our view that if appropriately developed and approved for human use, curcumin or more potent derivatives could potentially be used with artesunate in the future. An open-labelled study to evaluate the safety of future curcumin formulations, combined with intravenous artesunate, examining as endpoints neurological sequelae or survival rate, should be carried out in future. Since treatment for CM is initiated after the onset of coma in most cases, it will be important to evaluate whether curcumin can reverse or shorten the comatose period and/or reduce the risk of neurological sequelae. We anticipate that due to its specific anti-malarial activity, it is unlikely that curcumin will exacerbate the evolution of CM.

### Limitations for the use of curcumin in the clinic

Although the information discussed above suggests a plausible and beneficial use of curcumin in the management of CM, an important limitation hindering the clinical advancement of this promising molecule is its poor ADME properties. Curcumin shows low oral bioavailability, high tissue distribution, rapid metabolism and elimination [57,82]. The low bioavailability of curcumin is in part due to its hydrophobic nature and when administered orally, curcumin undergoes conjugation leading to the formation of curcumin glucuronide and sulfates in the intestinal wall and the liver [35]. Numerous strategies are currently being investigated to enhance curcumin’s bioavailability. One of these approaches involved the use of a bioavailability enhancer such as piperine from black pepper. Piperine is an inhibitor of hepatic and intestinal glucuronidation which has enhanced the bioavailability of curcumin when administered in animal and human volunteers [83]. Other attempts to increase curcumin’s bioavailability include liposomal curcumin [84], curcumin nanoparticles and curcumin-phospholipid complexes [85]. Thus, a successful enhancement of curcumin bioavailability that preserves its safety in humans is likely to bring this promising natural product to the forefront of therapeutic agents for the treatment of various conditions including malaria.

## Concluding remarks

Curcumin has a long history of therapeutic use in the Ayurvedic and Chinese traditional medicine and multiple clinical trials are ongoing to evaluate its efficacy in the management of various human disorders including cancer, neurodegenerative diseases and diabetes [56] . Curcumin is considered a safe compound by the United States Food and Drug Administration and is commercially available as a dietary supplement (Figure 1). Curcumin is well-tolerated in humans and with respect to the management of CM might exert its therapeutic effects by inhibiting NF-κB activation, followed by downregulation of proinflammatory cytokine production and expression of cytoadhesion molecules on endothelial cells. The fact that cytoadherence of the malaria parasite continues long after parasites have been killed by anti-malarial drugs, supports the development of adjunctive therapies to reverse the pathophysiological consequences of cytoadherence [86]. With extensive research efforts ongoing to explore the clinical applications of curcumin in chronic inflammatory disorders, diabetes and cancer, the development of oral and parenteral curcumin formulations or curcumin analogues with improved bioavailability while retaining their immunomodulatory properties and possibly more potent anti-malarial activity deserves investigation. Drug discovery efforts focused on molecules with dual, immunomodulatory and anti-parasitic action, may pave the way for their use as an adjunctive therapy for the management of uncomplicated and severe malaria.

## Competing interests

The authors declare that they have no competing interests related to this work.

## Authors’ contributions

PNM drafted the manuscript. DT and LV participated in the writing and made corrections to the manuscript. All the authors read and approved the final version.

## Acknowledgements

Financial support: This work was supported by a grant from the European Commission Sixth Framework Programme FP6- IP-18834, AntiMal to DT from the University of Milan and LV from the London School of Hygiene and Tropical Medicine. PNM is recipient of a studentship from the AntiMal International PhD programme, an EMBL collaborative training programme. The European Commission had no role in study design, data collection, analysis or interpretation, the writing of the manuscript, or the decision to submit the work for publication.
